# A Multi-Domain Feature Fusion CNN for Myocardial Infarction Detection and Localization

**DOI:** 10.3390/bios15060392

**Published:** 2025-06-17

**Authors:** Yunfan Chen, Jinxing Ye, Yuting Li, Zhe Luo, Jieqiang Luo, Xiangkui Wan

**Affiliations:** 1 Hubei Key Laboratory for High-Efficiency Utilization of Solar Energy, Hubei University of Technology, Wuhan 430068, China; yfchen@hbut.edu.cn (Y.C.);; 2 School of Computer Science, Hubei University of Technology, Wuhan 430068, China; ytli@hbut.edu.cn; 3 School of Artificial Intelligence, Shenzhen Polytechnic University, Shenzhen 518055, China; 4 Puleap Health Technology Co., Ltd., Guangzhou 510710, China; luojieqiang@puleap.com

**Keywords:** deep learning, myocardial infarction, multi-domain features, ECG

## Abstract

Myocardial infarction (MI) is a critical cardiovascular disease characterized by extensive myocardial necrosis occurring within a short timeframe. Traditional MI detection and localization techniques predominantly utilize single-domain features as input. However, relying solely on single-domain features of the electrocardiogram (ECG) proves challenging for accurate MI detection and localization due to the inability of these features to fully capture the complexity and variability in cardiac electrical activity. To address this, we propose a multi-domain feature fusion convolutional neural network (MFF–CNN) that integrates the time domain, frequency domain, and time-frequency domain features of ECG for automatic MI detection and localization. Initially, we generate 2D frequency domain and time-frequency domain images to combine with single-dimensional time domain features, forming multi-domain input features to overcome the limitations inherent in single-domain approaches. Subsequently, we introduce a novel MFF–CNN comprising a 1D CNN and two 2D CNNs for multi-domain feature learning and MI detection and localization. The experimental results demonstrate that in rigorous inter-patient validation, our method achieves 99.98% detection accuracy and 84.86% localization accuracy. This represents a 3.43% absolute improvement in detection and a 16.97% enhancement in localization over state-of-the-art methods. We believe that our approach will greatly benefit future research on cardiovascular disease.

## 1. Introduction

According to statistics from the World Health Organization, the number of people affected by cardiovascular diseases (CVDs) and the proportion of deaths they cause increase annually. CVDs are now the leading cause of death worldwide, resulting in substantially more deaths and fatalities than cancer and other diseases [1]. The high prevalence, disability, and mortality rates are the main characteristics of CVDs [2]. Common CVDs include hypertension, hyperlipidemia, angina pectoris, coronary heart disease, and myocardial infarction (MI). MI, commonly known as a heart attack, is the leading cause of death from cardiovascular disease. It is characterized by its sudden onset, critical condition, high mortality rate, and potential for serious complications. Early diagnosis and accurate treatment are crucial for the management and prognosis of MI [3], substantially reducing complications and greatly improving patient survival rates [4].

The electrocardiogram (ECG) is a crucial tool for recording cardiac signals, objectively reflecting the physiological status of different parts of the human heart to a great extent. Due to its accessibility and non-invasiveness, the ECG is commonly used for clinical MI detection and localization [5]. Traditionally, the identification of MI relied heavily on manual ECG interpretation by experts, a process that is often time-consuming and, given the lengthy diagnostic sessions, susceptible to human error. However, with advancements in computer science and signal processing technology, research on the automatic detection of MI has increased substantially, leveraging traditional machine learning and deep learning methods to potentially mitigate these limitations.

Traditional machine learning-based MI detection methods require the manual extraction of ECG features, which are then classified by classifiers, such as support vector machine (SVM) [6,7,8,9,10,11], k-nearest neighbor (KNN) [7,8,12], and decision tree (DT) [7]. Arif et al. [13] used the discrete wavelet transform (DWT) to extract features from a 12-lead ECG and employed the KNN classifier to detect and locate MI. Dohare et al. [9] extracted 220 features from the ECG, including P-wave, QRS, ST-T complex, and QT interval, and utilized principal component analysis (PCA) to select 14 input features in an SVM model for MI detection. However, manual feature extraction has substantial limitations, potentially reducing the classifier’s accuracy. Additionally, obtaining useful ECG features remains challenging and heavily relies on clinical diagnostic experience. The lack of important information during manual feature extraction may also lead to misdiagnosis [14].

In contrast, deep learning-based MI detection methods are automatic and do not require manual analysis and feature extraction. Wang et al. [15] proposed a multi-lead integrated neural network approach that combined a three-seeded network with multi-lead ECG signals for MI detection. Feng et al. [16] combined convolutional neural networks (CNNs) and long short-term memory (LSTM) to learn MI features. Xiong et al. [17] used a multi-lead neural network based on DenseNet to localize MI with a 12-lead ECG. Strodthoff et al. [18] employed a fully connected neural network to detect MI. Qu Jierui et al. [19] proposed an interpretable shapelet-based approach that combines dynamic learning with deep learning to capture the intrinsic dynamics of ECG and extract substantial shapelets. Zhang et al. [20] adopted ensemble learning methods to detect MI by extracting energy entropy and morphological features from ECG signals. The above deep learning-based MI detection methods use 1D ECG signals as input. However, 1D ECG signals provide only single-point time and amplitude information, which cannot fully reveal the spatial distribution characteristics of cardiac electrical activity. In contrast, 2D ECG signals provide the time and amplitude information and the overall change trend of the signal.

Recent studies have proposed various approaches using 2D ECG images as inputs for MI detection. Huang et al. [21] and Rahhal et al. [22] explored the use of time-frequency images generated by short-time Fourier transform (STFT) or continuous wavelet transform (CWT) as 2D inputs, achieving substantially improved ECG classification results compared with traditional 1D ECG analysis. Hao et al. [23] employed 12-lead ECG images in a multi-branch fusion network for MI detection. Swain et al. [24] introduced an enhanced Stockwell transform and phase distribution mode for automatic MI detection. Zhang et al. [25] applied the Gramian Angular Field (GAF) method to convert ECG time series into images, then feature extraction using a PCA network. Yousuf et al. [26] proposed using 2D CNNs for detecting MI by transforming ECG signals into grayscale images.

Existing deep learning-based methods for MI detection and localization have shown promising results. However, many of these methods focus exclusively on the time domain characteristics of ECG signals, neglecting their frequency domain features. In practical applications, disease diagnosis cannot rely solely on single-domain features; integrating multiple types of features becomes imperative. Combining information from both time and frequency domains is essential to enhance the model’s generalization ability. To achieve this, ECG signals can be decomposed into multiple scales, facilitating the generation of time-frequency images across various frequency ranges. These images enable a detailed exploration of the complexity and dynamics of cardiac electrical activity, thereby empowering MI detection and localization algorithms to leverage both types of features effectively and improve overall accuracy.

Therefore, this study proposes a multi-domain feature fusion convolutional neural network (MFF–CNN) that integrates ECG signals’ time, frequency, and time-frequency domain features for MI detection and localization. The main contributions are summarized below.

• The effective generation of frequency-domain spectrum and time-frequency images, combined with time-domain ECG signals, creates multi-domain information inputs. Leveraging S-transform for time-frequency representation and GAF for spectral analysis, this approach significantly enhances feature representation and generalization ability, thereby improving MI detection and localization accuracy.
• Introduction of a novel MFF–CNN for automatically extracting deep features from input ECG signals, spectrum images derived from GAF, and time-frequency images generated by S-transform. This model integrates complementary characteristics from each domain, leveraging the strengths of both S-transform and GAF in capturing MI-related features, enabling effective multi-domain feature learning for MI detection and localization.
• The MFF–CNN achieves superior results compared with existing methods, demonstrating its effectiveness in detecting and localizing MI. On the well-known PTB diagnostic ECG database, our proposed method sets a new benchmark in inter-patient evaluation with a detection accuracy of 99.98% and a location accuracy of 84.86%. Furthermore, in generalization tests using the PTB data for training and the PTB-XL data for validation, the MFF–CNN obtains a location accuracy of 78.21%, far surpassing that of single-domain models.

The remainder of this paper is organized as follows: Section 2 describes the data preprocessing, GAF, S transform, and the proposed MFF-CNN. Section 3 presents the experimental results. Section 4 discusses the experimental results. Finally, Section 5 provides the conclusions.

## 2. Materials and Methods

**Figure 1:**
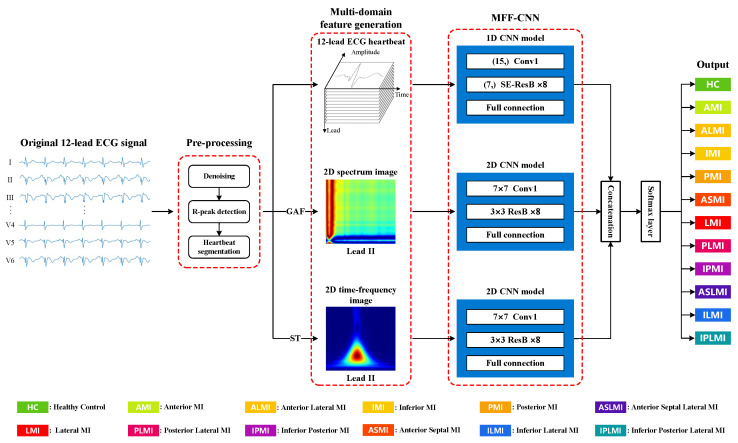
The overall structure of the proposed MI detection and localization method.

Figure 1 illustrates the structure of the proposed MI detection and localization method. The 12-lead ECG signal initially undergoes preprocessing and segmentation into individual heartbeats across all 12 leads. The spectrum image (using the GAF method) and the time-frequency image (via the S transform) are derived from lead II of these segmented heartbeats. Lead II is critical for ECG diagnosis as it aligns with the heart’s electrical axis, clearly showing atrial and ventricular depolarization, and typically has less noise interference, ensuring better quality for GAF and S-transform analysis. Subsequently, the obtained images and the 12-lead heartbeat data train a 2D CNN network and a separate 1D CNN network. Ultimately, the outputs from these networks are integrated to determine the precise location and type of MI. By integrating multi-domain features inherent in ECG signals, this approach substantially improves the accuracy and robustness of MI localization.

### 2.1. Data Preprocessing

This study utilizes the PTB diagnostic ECG database [27] and PTB-XL dataset [28] from the Physikalisch-Technische Bundesanstalt of the German National Metrology Institute. The PTB database serves as an open-source resource for MI diagnosis. It comprises 549 records from 290 subjects, each with one or more records. The subjects’ ages range from 17 to 87 years, with an average age of 57.2. The male-to-female ratio is 2.58:1. Each record has a sampling rate of 1000 Hz and includes 15 channels: the standard 12-lead ECG signals (I, II, III, aVR, aVL, aVF, and V1–V6) and the 3 Frank ECG signals (VX, VY, and VZ). The database lacks beat-by-beat annotations but provides detailed clinical summaries and other pertinent information in the header files of most ECG recordings. It encompasses 148 patients with MI and 52 healthy control subjects. Among the 148 MI patients, 21 were not annotated with the specific infarction region; thus, records from these 21 MI patients were excluded from the study. Table 1 summarizes the samples from the PTB database used in this research.

The PTB-XL ECG dataset consists of 21,837 clinical 12-lead ECG records from 18,885 patients. Each record is 10 s long and has a sampling rate of 500 Hz. Cardiologists have annotated the data covering 71 types of heart disease, with multiple annotations possible in most records. This study provides an overview of the PTB-XL dataset, which includes 2184 normal records and 5469 records depicting nine types of MI, detailed in Table 2. Due to the differing sampling rates between the PTB-XL dataset and the PTB database, we resampled the PTB-XL dataset to 1000 Hz to ensure consistency in frequency across both databases.

The ECG signal must be preprocessed to remove noise sources, such as myoelectric interference, baseline drift, and industrial frequency interference to ensure accurate classification results. This involves eliminating noise signals from the original signal before analysis to prevent interference from affecting the results. The initial step involves baseline wander removal via median filtering, which suppresses low-frequency drift caused by respiration or movement by vertically shifting the signal to zero-center the isoelectric line. Subsequently, the improved threshold wavelet denoising method [29] separates the ECG signal from noise, as depicted in Equation (1).

(1) $${\hat{d}}_{b}\left(c\right)=\left\{\begin{array}{c} \mathrm{sgn}\left(d_{b}\left(c\right)\right)\left(\left|d_{b}\left(c\right)\right|-TE_{b}\right),\left|d_{b}\left(c\right)\right|>TE_{b}\\ 0\;,\left|d_{b}\left(c\right)\right|\leq TE_{b} \end{array}\right.\;$$

where *b* (*b* = 1,...,9) represents the number of levels of wavelet decomposition, and *c* denotes the number of sample points in the signal. $TE_{b}$ is the set threshold, calculated as $TE_{b}=\left(\sigma_{b}\sqrt{\left(2\log∥d_{b}∥\right)}\right)/\log\left(b+1\right)\;$, where $∥d_{b}∥$ denotes the $L_{2}$ norm (Euclidean norm) of the detail coefficients at the *b*-th level of the wavelet decomposition, $\sigma_{b}$ denotes the estimated noise level calculated as $\sigma_{b}=\left(median(\left|d_{b}\right|)\right)/0.6745$ [30], and 0.6745 is the value corresponding to 75% of the area of under a standard Gaussian distribution with mean 0 and variance 1 (*p* = 0.25).

**Figure 2:**
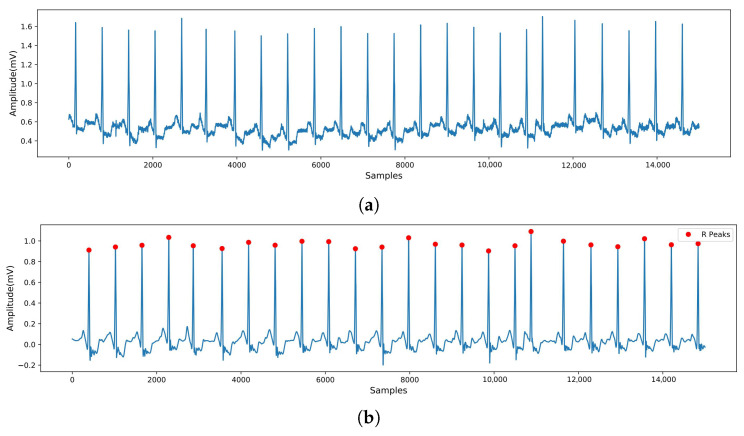
ECG signals before and after preprocessing. (**a**) Original ECG signal. (**b**) ECG signal after denoising.

The dB6 wavelet was selected as the wavelet basis due to its similarity in morphology to the ECG signal. A 5-level wavelet decomposition was applied to the signal, and coefficients smaller than $TE_{b}$ were thresholded to zero to eliminate additional noise signals. The Pan–Tompkins algorithm [31] was utilized to localize QRS wave clusters and R peaks. Each heartbeat was defined as 250 sample points before the R peak and 400 sample points after the R peak, resulting in 651 sample points per beat. Figure 2 illustrates a comparison of the ECG signals before and after processing.

### 2.2. Gramian Angular Field for Spectrum Image Generation

In this study, a discrete Fourier transform was employed to compute the spectrum of the ECG signal. Subsequently, the GAF method [32] was utilized to transform the 1D spectrum into a 2D image, leveraging its advantage in preserving temporal dependencies and facilitating feature extraction in the frequency domain. The algorithm consists of three steps. First, any 1D signal $X=\left(x_{1},x_{2},...,x_{n}\right)$ is rescaled to fit within the interval [−1,1] using Equation (2).

(2) $$\tilde{x_{i}}=\frac{\left[x_{i}-max\left(X\right)\right]+\left[x_{i}-min\left(X\right)\right]}{max(X)-min(X)}$$

The sequence *X* is transformed and represented in polar coordinates. Each value is encoded as an angular cosine $\varphi_{i}$ and a radius $r_{i}$, which are calculated using Equation (3).

(3) $$\left\{\begin{array}{c} \varphi_{i}=\arccos\left(\tilde{x_{i}}\right),-1\leq \tilde{x_{i}}\leq 1,\tilde{x_{i}}\in \tilde{X,}\\ r_{i}=\frac{t_{i}}{N},t_{i}\in N,i=1,2,...,n, \end{array}\right.$$

The formula uses $t_{i}$ to denote the timestamp and *N* as a constant factor. Representing the time series in polar coordinates offers a novel approach. As time progresses, values shift among various points on the circular representation. Converting the adjusted time series into polar coordinates facilitates leveraging the angular perspective by computing the angular difference between consecutive points. This aids in identifying the temporal correlations across different intervals. Equation (4) subsequently defines the GAF.

(4) $$\begin{array}{cc} G & =\left[\begin{array}{ccc} \sin(\varphi_{1}-\varphi_{1}) & \ldots & \sin(\varphi_{1}-\varphi_{n})\\ \sin(\varphi_{2}-\varphi_{1}) & \ldots & \sin(\varphi_{2}-\varphi_{n})\\ \vdots & \ddots & \vdots \\ \sin(\varphi_{n}-\varphi_{1}) & \ldots & \sin(\varphi_{n}-\varphi_{n}) \end{array}\right]\\ \; & ={\sqrt{I-diag(\tilde{X}⊙\tilde{X})}}^{\prime }\times \tilde{X}-{\tilde{X}}^{\prime }\times \sqrt{I-diag(\tilde{X}⊙\tilde{X}}) \end{array}$$

Equation (4) illustrates that *I* is a unit row vector and *G* represents a Gram matrix. In Equation (4), the Gram matrix G has zero diagonal elements by construction, as $sin(\varphi_{i}-\varphi_{i})=0$. This reflects the elimination of self-difference terms and focuses attention on pairwise temporal dependencies. While G is thus not positive definite, its semidefinite structure preserves critical phase-based relationships for feature extraction. Transforming from 1D to 2D features provides two primary advantages: smoothing the spectral sequence through piecewise aggregation approximation [33] to maintain crucial temporal trends for CVDs analysis and enhancing the intuitive representation of CVDs-induced spectral changes. This leverages the capabilities of 2D deep learning in computer vision for precise feature identification.

**Figure 3:**
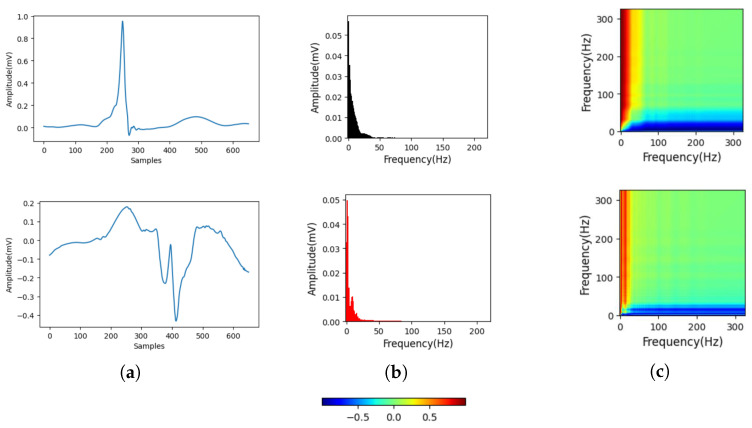
ECG spectral signals of MI and HC after GAF transformation are presented in the images. (**a**) HC and MI ECG without noise. (**b**) 1D Spectrum of HC and MI. (**c**) Spectrum image of HC and MI.

Figure 3 illustrates the transformation process of 1D spectral images of ECG signals into 2D spectral images using the GAF method for both MI and HC subjects, with the upper half depicting ECGs from HC subjects and the lower half from MI subjects. The ECG signals of MI subjects exhibited more low-frequency signals and a less concentrated overall frequency distribution than HC subjects. However, these changes were not evident in the 1D spectra. The GAF method was employed to convert the signals to images, enhancing visibility and making the changes induced by MI more pronounced and apparent.

### 2.3. S Transform

The S transform (ST) generalizes the CWT [34], providing high-frequency resolution, no cross-terminal interference, strong noise immunity, and an adjustable window function [35]. Equation (5) defines the ST of the signal x(t).

(5) $$S\left(\tau ,f\right)=\int_{-\infty }^{\infty }x\left(t\right)\frac{\left|f\right|}{\sqrt{2\pi }}e^{-\frac{{\left(\tau -t\right)}^{2}f^{2}}{2}}e^{-i2\pi ft}dt$$

where τ represents the time shift factor and *f* denotes frequency. The ST is utilized in ECG analysis to offer precise time-frequency information. Initially proposed by Davis and Mermelstein in 1980, this transform employs an adjustable Gaussian window that effectively accommodates frequency variations in the signal. Compared with the STFT, the ST provides superior time-frequency resolution. Given the temporal variability in the heart’s electrical activity, the Gaussian window with inverse frequency dependency accurately captures this dynamic property.

Additionally, the ST provides superior phase resolution, enabling the detection of even minor signal changes. Due to the complexity and noise often present in ECG signals, a tool with excellent phase resolution is crucial for extracting valuable information. The ST, with its phase correction and scalable Gaussian window, facilitates the precise extraction of the heart’s electrical activity features and provides an accurate time-frequency representation. The inverse frequency dependence of the Gaussian window allows finer capture of high-frequency signal components, transient feature detection, and estimation of the signal’s time-frequency distribution. This enhances our understanding of the heart’s dynamic characteristics and abnormal manifestations, offering valuable clinical insights for diagnosis and treatment.

**Figure 4:**
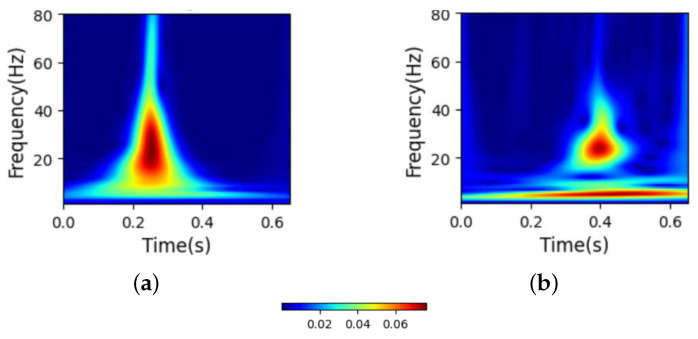
Images of heartbeats from MI and HC subjects after S transform. (**a**) ST image of HC. (**b**) ST image of MI.

Figure 4 shows the ST images of the heartbeats of MI and HC subjects. The ST image in Figure 4b indicates that, compared with HC subjects, the main frequency of MI subjects appears at 0.4 s, and the overall frequency distribution is not concentrated, with a substantial number of low-frequency signals present throughout the entire heartbeat.

### 2.4. MFF–CNN Network

**Figure 5:**
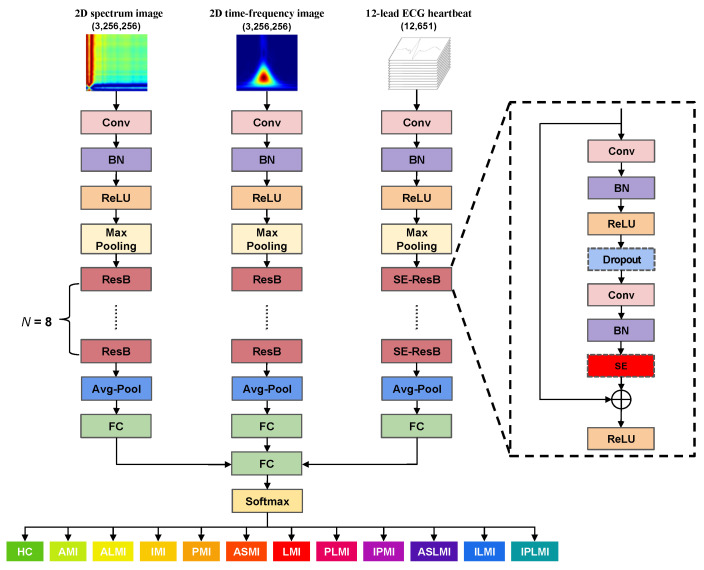
Architecture of the proposed MFF–CNN network.

Figure 5 depicts the architecture of the proposed MFF–CNN, which incorporates the standard ResNet18 network and the optimized SE-ResNet18 network. The ResNet18 network is the primary structure of the model due to its excellent balance between performance and efficiency, robust feature extraction capabilities, broad applicability across various tasks, and ease of deployment and debugging. Due to the variability in data dimensions, we utilized the classical ResNet18 network to process 2D data. To adapt to the characteristics of 1D ECG data, we converted the 2D ResNet18 network into a 1D ResNet18 network and adjusted the initial 7 × 7 convolutional kernel to a 1D convolutional kernel of size 15. This adjustment is because larger convolutional kernels can help the network learn more meaningful features. Additionally, the subsequent 3 × 3 convolutional kernel was replaced with a 1D convolutional kernel of size 7.

To enhance the network’s ability to characterize features, we added a squeeze and excitation (SE) block structure to each residual block (ResB) [36]. The SE block enhances the representation of feature channels through its two core operations: “Squeeze” and “Excitation”. The “Squeeze” operation summarizes channel-wise features via global average pooling, creating a global description. Meanwhile, the “Excitation” operation utilizes a Multi-Layer Perceptron to evaluate the importance of each channel based on this global information, outputting a weight vector. This modification enables the model to effectively integrate global information with channel significance, subsequently bolstering its performance, robustness, and adaptability to a wide range of complex scenarios by learning and emphasizing crucial weights and features across different channels. Additionally, a dropout layer with a dropout rate of 0.2 was added between the two convolutional kernels of each ResB to reduce the risk of overfitting. These enhancements improve the model’s ability to adapt to data of varying dimensions, resulting in more accurate feature extraction and classification.

Finally, we effectively fuse and integrate the output information from each network by concatenating the feature vectors from different input sources to form a combined feature vector, which serves as the input to the fully connected layer. Subsequently, a softmax layer normalizes the output of the fully connected layer to obtain the final classification results.

This strategy leverages the strengths of each network and improves the model’s classification performance. Table 3 presents the parameters of the two types of networks used in the experiment.

## 3. Experimental Results

In this section, we evaluate and compare the MI detection and localization performance of the proposed MFF–CNN model with three single-domain models: the 1D ECG model, the 2D spectrum image model, and the 2D time-frequency image model. The evaluation is conducted on the PTB database using both intra-patient and inter-patient paradigms. The generalizability of the proposed MFF–CNN model is further verified using the PTB-XL dataset.

### 3.1. Experimental Settings

During the training process, all models were trained using the cross-entropy loss function. Stochastic gradient descent with a learning rate of 0.001 and momentum of 0.9 was employed for parameter updates. The model’s loss is minimized through iterative steps, and its parameters are updated using error back-propagation. The batch and epoch sizes were set to 64 and 60, respectively. The experiments we conducted using Python 3.7. The deep learning program ran on the PyTorch 1.10.1 framework, utilizing NVIDIA GeForce RTX 4060 laptop GPUs (Nvidia, Santa Clara, CA, USA) to accelerate the training process. The methodology was implemented on a PC with a 5.40 GHz Intel Core i9-13900HX CPU (Intel, Santa Clara, CA, USA), 16 GB RAM, and Windows 11 operating system (Microsoft, Redmond, WA, USA).

### 3.2. Evaluation Indicators

To facilitate objective and quantitative comparisons of classification performance, we evaluated the classification results using accuracy (Acc), sensitivity (Sen), precision (Pre), specificity (Spe), and F1-score (F1). These metrics were defined as follows:

(6) $$Acc=\frac{TP+TN}{TP+FP+TN+FN},$$

(7) $$Sen=Recall=\frac{TP}{TP+FN},$$

(8) $$Pre=\frac{TP}{TP+FP},$$

(9) $$Spe=\frac{TN}{TN+FP},$$

(10) $$F_{1}-\mathrm{Score}=\frac{2\times Sen\times Pre}{\left(Sen+Pre\right)}$$

where TP and TN denote the number of true-positive and true-negative heartbeats, respectively. FN and FP represent the number of false-negative and false-positive heartbeats. To assess the overall accuracy of the model in localizing MI, we define the model’s overall classification accuracy as $Acc_{T}$, calculated using Equation (11).

(11) $$Acc_{T}=\frac{\sum_{y=1}^{12}TP_{y}}{\sum_{y=1}^{12}TP_{y}+FN_{y}}$$

where $TP_{y}$ represents the number of correctly detected heartbeats of each type, while $FN_{y}$ is the number of each type of heartbeat that was not correctly diagnosed.

### 3.3. Intra-Patient Evaluation of the Performance of MI Detection and Localization on the PTB Database

For the intra-patient experiments, the recorded heartbeats were randomly divided into training, validation, and test sets in a ratio of 7:2:1. The proposed dataset splitting strategy substantially reduces computational expenses compared with K-fold cross-validation. Additionally, the fixed validation and test sets provide a stable and consistent basis for evaluating the performance of different models. The presented results reflect all the experimental outcomes obtained from the entire PTB database.

Table 4 presents the experimental results of each model for MI detection in the intra-patient paradigm. Table 5 presents the 10-fold cross-validation results of the MFF–CNN model for detecting MI under the intra-patient paradigm. Under this paradigm, single-domain models using 12-lead ECG signals, GAF images, and ST images as inputs have all demonstrated satisfactory performance in MI detection. However, the MFF–CNN model achieved superior performance with an accuracy of 99.99%, sensitivity of 100%, precision of 99.99%, specificity of 99.97%, and F1-score of 100%, surpassing all single-domain models.

Similarly, as depicted in Table 6, our MFF–CNN model exhibits higher accuracy and sensitivity than all single-domain models for MI location. Although the precision and F1-score of the MFF–CNN model are slightly lower than those of the single-domain ST images model, the overall performance of the MFF–CNN is superior.

**Figure 6:**
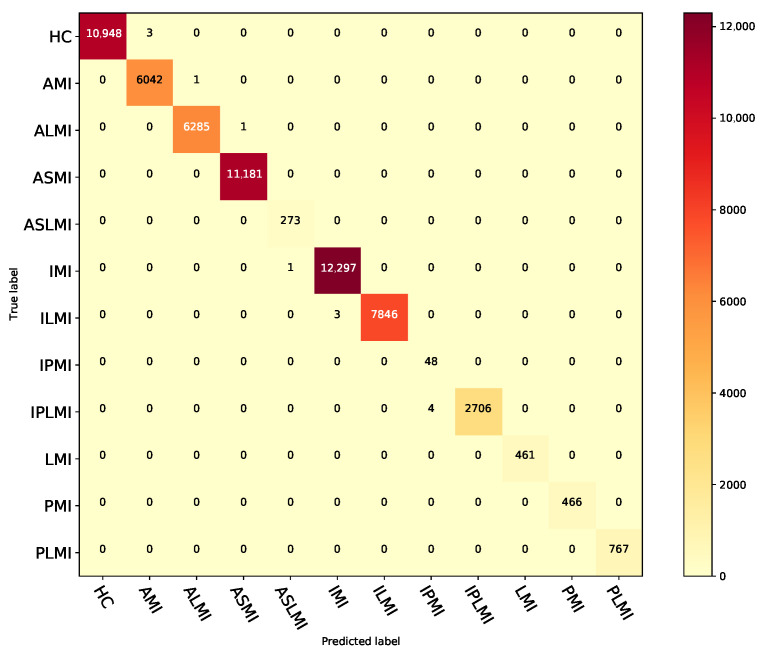
Confusion matrix for the fusion model under the intra-patient paradigm on the PTB database.

Figure 6 illustrates the confusion matrix of the fusion model in the intra-patient paradigm of the PTB database for MI localization. Table 7 presents the fusion model’s performance in localizing different types of MI under this paradigm.

### 3.4. Inter-Patient Evaluation of the Performance of MI Detection and Localization on the PTB Database

For the inter-patient experiments, we randomly divided all records into a training set and a test set in a 7:3 ratio based on the number of patients. However, the validation set was insufficient for further optimization due to the limited number of samples in specific MI categories. Therefore, we opted for a straightforward 7:3 division between the training and testing sets without establishing a separate validation set. Additionally, since the PTB database contains instances from individual patients, we sourced the corresponding patient data from the PTB-XL dataset to facilitate the completion of the inter-patient experiments.

Using different patients for training and testing data increases the complexity of MI detection and localization. The results presented reflect the model’s predictions on the test set. As shown in Table 8, the proposed MFF–CNN demonstrates impressive performance in MI detection, achieving an accuracy of 99.98%, a sensitivity of 100%, a precision of 99.97%, a specificity of 99.95%, and an F1 score of 99.98%, surpassing all single-domain models. Table 9 presents the 10-fold cross-validation results of the MFF–CNN model for detecting MI under the inter-patient paradigm.

Regarding MI localization, the MFF–CNN model outperforms the single-domain model in accuracy and sensitivity under the same paradigm, as displayed in Table 10. The MFF–CNN model achieved high-precision localization under the more complex inter-patient paradigm, with an overall accuracy of 84.86%, a sensitivity of 62.90%, a precision of 64%, a specificity of 98.60%, and an F1-score of 60.59%. These results demonstrate the model’s stability and accuracy on inter-patient data.

In the PTB inter-patient experiments, multi-domain feature fusion resulted in a substantial improvement in MI detection and localization compared with models relying solely on single-domain features. The need for the model’s generalization ability becomes more critical as the difference between the training and testing data increases in the inter-patient paradigm.

**Figure 7:**
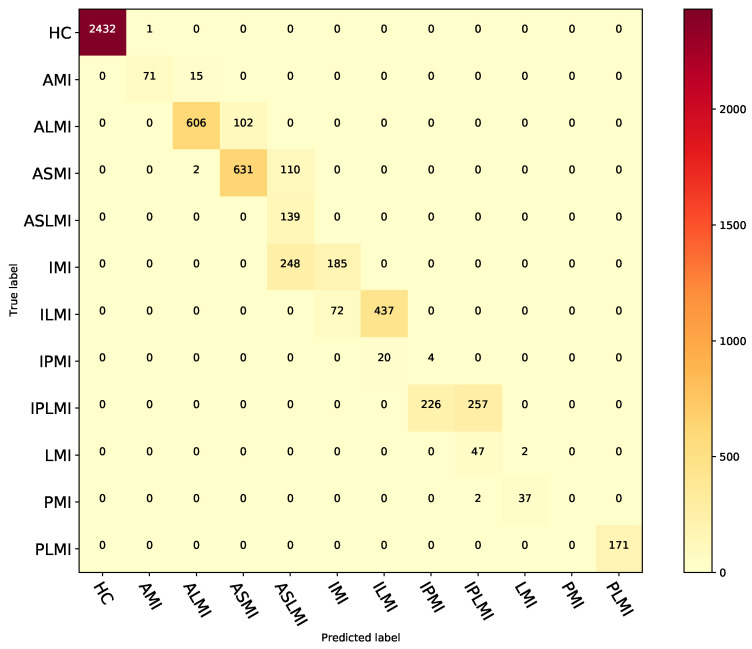
Confusion matrix for the fusion model under the inter-patient paradigm on the PTB database.

The experimental results indicate that the proposed fusion model, MFF–CNN, is more effective than single-domain models in detecting and localizing MI under the inter-patient paradigm. This is due to the fusion model’s ability to comprehensively utilize feature information from different domains and perspectives, resulting in more accurate disease pattern identification and stronger stability and generalization. Figure 7 shows the confusion matrix of the fusion model for MI localization under the inter-patient paradigm for the PTB database. Table 11 shows the performance of the fusion model in localizing different types of MI in the inter-patient paradigm of the PTB database.

### 3.5. Ablation Experiments Under the Inter-Patient Paradigm

To assess how time-domain, frequency-domain, and time-frequency-domain ECG features affect MI localization, we conducted ablation experiments. By systematically removing or combining features from different domains, we analyzed their contributions to the model’s MI localization accuracy. Since data partitioning under the intra-patient paradigm may bias model performance evaluations in ablation studies, we uniformly used inter-patient partitioning in ablation experiments to ensure reliable results.

As shown in Table 12, under the inter-patient paradigm of the PTB dataset, the impact of different domain feature combinations on the myocardial infarction localization model performance exhibits significant variations. When using single-domain features, ECG signals alone achieved an Acc of 56.37% and Sen of 49.27%, showing preliminary localization capability with room for improvement. Switching to GAF images increased Acc to 58.65% and Sen to 52.53%, indicating complementary information. ST images delivered the best single-domain performance with Acc reaching 61.66% and Sen 55.37%, demonstrating superior information representation. Multi-domain feature fusion significantly enhanced performance: ECG+GAF achieved Acc 68.42% and Sen 56.21%, while ECG+ST reached Acc 72.15% and Sen 59.83%, highlighting ST’s critical contribution. Notably, the GAF+ST combination underperformed with Acc 65.33% and Sen 53.97%. The tri-modal fusion of ECG+GAF+ST yielded optimal results—Acc 84.86% and Sen 62.96%—proving the necessity and substantial advantage of multi-domain feature fusion in improving MI localization accuracy and effectiveness.

### 3.6. Generalizability Evaluation of the Proposed Method on the PTB-XL Dataset

Given the model’s generalization ability and importance in practical applications, we selected the PTB-XL dataset as the test dataset for verification. It is important to note that the PTB-XL dataset was not used in the model’s training process but was only utilized during the testing phase. This approach ensures an objective and accurate evaluation of the model’s generalization performance across various datasets.

In the experiments on the PTB-XL dataset, the fusion model MFF–CNN demonstrated excellent MI detection performance, outperforming all single-domain models. As shown in Table 13, the fusion model achieved a high accuracy of 91.57% for MI detection on the PTB-XL dataset, with a perfect sensitivity of 100% and a high precision level of 88.73%. Although the specificity was slightly lower at 74.93%, the F1 score was as high as 94.03%. Compared with its performance on the PTB database, the model’s performance on PTB-XL did not substantially decrease, demonstrating its strong generalization ability.

Table 14 shows that the fusion model MFF–CNN maintains good generalization ability for the task of MI localization across databases, with an accuracy of 78.21%, sensitivity of 73.95%, precision of 63.97%, specificity of 96.33%, and an F1-score of 61.49%. These results are substantially better than those of the single-domain models. Conversely, single-domain models exhibit poorer localization results. These results demonstrate that the fusion model can improve the accuracy and reliability of localization, while single-domain models have limitations in providing complete and accurate MI localization information.

Table 15 presents the fusion model’s performance in localizing different types of MI within the PTB-XL dataset.

### 3.7. Comparison Results with Existing Methods

Table 16 compares the proposed MFF–CNN with existing MI detection and localization methods based on the PTB database. In the intra-patient comparison, the proposed MFF–CNN achieves competitive results: 99.99% accuracy and 100% sensitivity for MI detection and 99.98% accuracy and 99.97% sensitivity for MI localization. While the 3D images [37] method reports 100% accuracy for MI detection, it does not address MI localization. In contrast, our method demonstrates superior overall performance and excels in detection and localization tasks. In inter-patient comparison, the accuracy of both MI detection and localization using our method surpasses that of all existing methods. Our approach enhances detection accuracy by 3.43% and improves localization accuracy by 16.97% compared with the most recent state-of-the-art method. This improvement is partly attributable to the heightened challenge posed by inter-patient studies on model generalization ability and partly due to the integration of frequency domain and time-frequency features of ECG signals, which many models lack, thereby reducing their generalization capability. In summary, the proposed method achieves outstanding performance in both MI detection and localization, with substantially higher accuracy in inter-patient scenarios than existing models.

## 4. Discussion

The experimental results clearly indicate that the proposed MFF–CNN fusion model outperforms traditional single-domain models in MI detection and localization, owing to its effective ability to integrate multi-domain features. While single-domain models are often limited to capturing either morphological and time-domain features or frequency-domain features, the MFF–CNN not only effectively combines both time and frequency information, but also utilizes the time-frequency analysis capabilities of the ST method to capture the instantaneous frequency characteristics that are pivotal for precise localization. This multi-faceted feature representation significantly boosts the model’s discriminative power, resulting in an overall MI localization accuracy of 84.86% in the inter-patient evaluation on the PTB database, a figure that markedly surpasses the 67.89% achieved by the current state-of-the-art method.

The proposed algorithm’s worst-case time complexity is O($n^{2}$), dominated by the GAF transformation stage. The GAF computes pairwise angular relationships on the time-series data, requiring O($n^{2}$) operations due to matrix constructions encoding temporal correlations. In contrast, the S-transform for time-frequency analysis exhibits an optimized complexity of O(h×nlogn), where *h* (the number of harmonic components) is typically small for ECG applications. Therefore, the S-transform complexity effectively scales as O(nlogn). Both the ResNet18 and SE-ResNet18 operate in constant time O(1), given their fixed input dimensions. Consequently, as the quadratic GAF term dominates the linear-logarithmic and constant terms, the overall algorithm complexity simplifies to O(n²)

Furthermore, the proposed MFF–CNN fusion model exhibits exceptional generalization ability. In the generalization evaluation on the PTB-XL dataset, characterized by heightened data complexity and diversity, single-domain models struggle to sustain their performance, achieving an overall accuracy of less than 30%. Conversely, the MFF–CNN’s proficiency in harnessing complementary information from multiple domains enables it to adapt more effectively to unforeseen data variations, maintaining a commendable overall accuracy of 78.21%. This robust performance underscores the significance of integrating multi-domain features in enhancing model generalization for complex medical signal automatic diagnosis tasks.

This study relied solely on Lead II data for training and testing the 2D CNN network. However, using a single lead may overlook the frequency and time-frequency relationships among the 12 leads, which are critical for capturing nuanced signal variations across different lead regions. In practical ECG signal processing, multi-lead data are more effective at capturing such variations, thereby providing richer diagnostic information. Future work will explore expanding the approach to incorporate all 12 leads for a more comprehensive analysis.

To address the limitations mentioned above, future research can be improved in two ways. Firstly, by collecting a more diverse and larger dataset of ECG signal data to improve its size and quality. Secondly, by adopting multi-lead data in 2D CNNs and combining them with more complex deep learning model structures, such as deeper convolutional neural networks or other advanced deep learning algorithms incorporating EMD-based feature extraction, to better capture and utilize the information in the ECG signal [45,46,47,48]. These improvements are expected to enhance the accuracy, generalizability, and practical applicability of the model.

## 5. Conclusions

This study proposes a fusion model, MFF–CNN, combining 1D ECG signals, 2D spectral images, and time-frequency images to explore potential MI detection and localization modalities. The model achieved good results under intra- and inter-patient paradigms in the PTB database. The proposed MFF–CNN model achieved 99.98% accuracy and 100% sensitivity for MI detection, 84.96% accuracy, and 62.90% sensitivity for MI localization under the PTB inter-patient paradigm. Furthermore, the model trained on the PTB database was evaluated using the PTB-XL dataset, demonstrating its good generalizability. The proposed method achieves 91.57% and 78.21% accuracy for MI detection and localization, respectively. Compared with previous studies, this indicates substantial potential for MI localization. We plan to acquire multi-lead ECG signals for a more comprehensive joint analysis in future studies. By combining various data types and leveraging deep learning techniques, we aim to develop a more efficient and accurate MI detection and localization model, providing robust support for medical diagnosis and treatment.

## Figures and Tables

**Table 1:** Summary of PTB database samples.

Types of MI	No. of Patients	No. of Records	No. of 12-Lead Heartbeats
Anterior MI (AMI)	17	47	6043
Anterior Lateral MI (ALMI)	16	43	6286
Anterior Septal MI (ASMI)	27	77	11,181
Anterior Septal Lateral MI (ASLMI)	1	2	273
Inferior MI (IMI)	30	89	12,298
Inferior Lateral MI (ILMI)	23	56	7849
Inferior Posterior MI (IPMI)	1	1	48
Inferior Posterior Lateral MI(IPLMI)	8	19	2710
Lateral MI (LMI)	1	3	461
Posterior MI (PMI)	1	4	466
Posterior Lateral MI (PLMI)	2	5	767
Healthy Control (HC)	52	80	10,951
Total	179	426	59,333

**Table 2:** Summary of PTB-XL dataset samples.

Types of MI	No. of Patients	No. of Records	No. of 12-Lead Heartbeats
Anterior MI (AMI)	286	290	2434
Anterior Lateral MI (ALMI)	181	208	1585
Anterior Septal MI (ASMI)	1780	2017	16,590
Inferior MI (IMI)	2055	2331	17,815
Inferior Lateral MI (ILMI)	350	394	2898
Inferior Posterior MI (IPMI)	26	30	218
Inferior Posterior Lateral MI(IPLMI)	49	50	424
Lateral MI (LMI)	125	135	1050
Posterior MI (PMI)	14	14	137
Healthy Control (HC)	1967	2184	21,854
Total	6833	7653	65,005

**Table 3:** Networks used in the experiment and their parameters.

Layer Name	ResNet18	SE-ResNet18
Conv1	7 × 7, 64, stride 2	(15,), 64, stride 2
Conv2_x	3 × 3, max pool, stride 2	(3,), max pool, stride 2
	$\left[\begin{array}{c} 3\times 3,64\\ 3\times 3,64 \end{array}\right]\times 2\;$	$\left[\begin{array}{c} \left(7,\right),64\\ \left(7,\right),64\\ fc,\left[4,64\right] \end{array}\right]\times 2$
Conv3_x	$\left[\begin{array}{c} 3\times 3,128\\ 3\times 3,128 \end{array}\right]\times 2\;$	$\left[\begin{array}{c} \left(7,\right),128\\ \left(7,\right),128\\ fc,\left[8,128\right] \end{array}\right]\times 2$
Conv4_x	$\left[\begin{array}{c} 3\times 3,256\\ 3\times 3,256 \end{array}\right]\times 2\;$	$\left[\begin{array}{c} \left(7,\right),256\\ \left(7,\right),256\\ fc,\left[16,256\right] \end{array}\right]\times 2$
Conv5_x	$\left[\begin{array}{c} 3\times 3,512\\ 3\times 3,512 \end{array}\right]\times 2\;$	$\left[\begin{array}{c} \left(7,\right),512\\ \left(7,\right),512\\ fc,\left[32,512\right] \end{array}\right]\times 2$
	Average pool, fc, softmax	Average pool, Dropout, fc, softmax

**Table 4:** Detection results of each model in the intra-patient paradigm on the PTB database.

Model Input	Acc (%)	Sen (%)	Pre (%)	Spe (%)	F1 (%)
ECG signal	99.98	99.98	99.98	99.94	99.98
GAF image	99.93	99.96	99.95	99.78	99.95
ST image	99.97	99.97	99.98	99.95	99.98
ECG signal & GAF image & ST image (MFF–CNN)	**99.99**	**100**	**99.99**	**99.97**	**100**

**Table 5:** 10-Fold Cross-Validation Results of the MFF–CNN Model for MI Detection under the Intra-Patient Paradigm.

Fold	Acc (%)	Sen (%)	Pre (%)	Spe (%)	F1 (%)
1	99.97	99.99	99.96	99.93	99.98
2	99.99	99.98	99.98	99.97	99.98
3	99.98	99.99	99.97	99.95	99.99
4	99.99	99.99	99.98	99.96	99.99
5	99.96	99.98	99.95	99.92	99.97
6	99.98	99.99	99.97	99.94	99.98
7	99.99	99.98	99.98	99.97	99.98
8	99.97	99.99	99.96	99.93	99.98
9	99.99	99.99	99.99	99.96	99.99
10	99.98	99.98	99.97	99.95	99.98

**Table 6:** Localization results of each model in the intra-patient paradigm of the PTB database.

Model Input	${\mathrm{Acc}}_{T}$ (%)	Sen (%)	Pre (%)	Spe (%)	F1 (%)
ECG signal	99.91	99.75	99.92	99.99	99.83
GAF image	99.65	99.74	99.53	99.96	99.63
ST image	99.91	99.94	**99.95**	99.99	**99.94**
ECG signal & GAF image & ST image (MFF–CNN)	**99.98**	**99.97**	99.32	**99.99**	99.63

**Table 7:** Performance of fusion model for MI Location under the intra-patient paradigm on the PTB database.

Class	Acc (%)	Sen (%)	Pre (%)	Spe (%)	F1 (%)
HC	99.99	99.97	100	100	99.99
AMI	99.99	99.98	99.95	99.99	99.97
ALMI	100	99.98	99.98	100	99.98
ASMI	100	100	99.99	100	100
ASLMI	100	100	99.64	100	99.82
IMI	99.99	99.99	99.98	99.99	99.98
ILMI	99.99	99.96	100	100	99.98
IPMI	99.99	100	92.31	99.99	96
IPLMI	99.99	99.85	100	100	99.93
LMI	100	100	100	100	100
PMI	100	100	100	100	100
PLMI	100	100	100	100	100
Average	99.99	99.97	99.32	99.99	99.63

**Table 8:** Detection results of each model in the inter-patient paradigm of the PTB database.

Model Input	Acc (%)	Sen (%)	Pre (%)	Spe (%)	F1 (%)
ECG signal	80.06	88.53	84.94	56.66	86.70
GAF image	73.18	91.59	76.51	22.31	83.37
ST image	79.19	90.00	83.07	49.35	86.40
ECG signal & GAF image & ST image (MFF–CNN)	**99.98**	**100**	**99.97**	**99.95**	**99.98**

**Table 9:** 10-Fold Cross-Validation Results of the MFF–CNN Model for MI Detection under the Inter-Patient Paradigm.

Fold	Acc (%)	Sen (%)	Pre (%)	Spe (%)	F1 (%)
1	99.97	100	99.96	99.92	99.98
2	99.99	99.99	99.98	99.97	99.99
3	99.98	100	99.97	99.95	99.99
4	99.99	99.99	99.98	99.96	99.98
5	99.96	99.98	99.94	99.92	99.97
6	99.98	100	99.97	99.94	99.98
7	99.99	99.99	99.98	99.97	99.99
8	99.98	99.99	99.96	99.93	99.97
9	99.99	99.98	99.99	99.97	99.99
10	99.98	99.99	99.97	99.96	99.98

**Table 10:** Localization results of each model in the inter-patient paradigm of the PTB database.

Model Input	${\mathrm{Acc}}_{T}$ (%)	Sen (%)	Pre (%)	Spe (%)	F1 (%)
ECG signal	56.37	49.27	55.36	96.11	55.32
GAF image	58.65	52.53	54.11	95.45	50.79
ST image	61.66	55.37	54.64	96.20	**63.89**
ECG signal & GAF image & ST image (MFF–CNN)	**84.86**	**62.96**	**64.03**	**98.68**	60.59

**Table 11:** Performance of fusion model for MI location under the inter-patient paradigm on the PTB database.

Class	Acc (%)	Sen (%)	Pre (%)	Spe (%)	F1 (%)
HC	99.98	99.96	100	100	99.98
AMI	99.72	82.56	99.98	98.61	89.87
ALMI	97.95	85.59	99.67	97.27	91.06
ASMI	96.32	84.93	97.99	86.08	85.5
ASLMI	93.85	100	93.69	27.97	43.71
IMI	94.5	42.73	98.66	71.98	53.62
ILMI	98.42	85.85	99.62	95.62	90.48
IPMI	95.77	16.67	96.1	1.74	3.15
IPLMI	95.27	53.21	99.08	83.99	65.15
LMI	98.56	4.08	99.36	5.13	4.55
PMI	99.33	0	100	0	0
PLMI	100	100	100	100	100
Average	97.47	62.96	98.68	64.03	60.59

**Table 12:** MI localization results of different combined models under the Inter-patient paradigm on the PTB dataset.

Model Input	Acc (%)	Sen (%)	Pre (%)	Spe (%)	F1 (%)
ECG signal	56.37	49.27	55.36	96.11	55.32
GAF image	58.65	52.53	54.11	95.45	50.79
ST image	61.66	55.37	54.64	96.20	63.89
ECG signal & GAF image	68.42	56.21	58.37	97.12	59.84
ECG signal & ST image	72.15	59.83	61.24	97.85	63.52
GAF image & ST image	65.33	53.97	57.89	96.78	57.12
ECG signal & GAF image & ST image (MFF–CNN)	**84.86**	**62.96**	**64.03**	**98.68**	**60.59**

**Table 13:** Detection results for each model on the PTB-XL dataset.

Model Input	Acc (%)	Sen (%)	Pre (%)	Spe (%)	F1 (%)
ECG signal	65.89	83.99	70.36	30.15	76.57
GAF image	71.95	86.66	74.98	42.92	80.40
ST image	66.51	85.30	70.46	29.41	77.17
ECG signal & GAF image & ST image (MFF–CNN)	**91.57**	**100**	**88.73**	**74.93**	**94.83**

**Table 14:** Localization results for each model on the PTB-XL dataset.

Model Input	${\mathrm{Acc}}_{T}$ (%)	Sen (%)	Pre (%)	Spe (%)	F1 (%)
ECG signal	28.73	12.90	12.95	91.14	14.99
GAF image	29.74	13.26	14.13	91.54	15.91
ST image	29.73	12.79	13.15	91.18	15.23
ECG signal & GAF image & ST image (MFF–CNN)	**78.21**	**73.95**	**63.97**	**96.33**	**61.49**

**Table 15:** Performance of fusion model for MI Location under the PTB-XL dataset.

Class	Acc (%)	Sen (%)	Pre (%)	Spe (%)	F1 (%)
HC	91.57	74.94	100	100	85.67
AMI	90.30	65.74	22.62	91.25	33.66
ALMI	98.00	90.28	55.55	98.19	68.78
ASMI	98.68	95.76	99.03	99.68	97.37
IMI	78.61	24.17	91.58	99.16	38.25
ILMI	76.29	32.99	6.63	78.31	11.03
IPMI	96.89	79.36	8.05	96.95	14.62
IPLMI	99.79	86.79	82.14	99.88	84.40
LMI	99.81	93.81	94.26	99.91	94.03
PMI	99.94	95.62	79.88	99.95	87.04
Average	92.99	73.95	63.97	96.33	61.49

**Table 16:** Comparing Novel Approach with Current Methods for MI Diagnosis.

Methods	Leads and Database	Detection or Location	Performance	
			Intra-Patient	Inter-Patient
CNN and BiLSTM [38]	12 leads PTB	Detection	Acc = 99.90% Se = 99.97% Sp = 99.54%	Acc = 93.08% Se = 94.42% Sp = 86.29%
CNN based on ResNet [39]	12 leads PTB	Detection and location	Detection: Acc = 99.92% Se = 99.98% Location: Acc = 99.72% Se = 99.63%	Detection: Acc = 95.49% Se = 94.85% Location: Acc = 55.74% Se = 47.58%
CNN and BiGRU with attention [40]	12 leads PTB	Detection and location	Detection: Acc = 99.93% Se = 99.99% Location: Acc = 99.11% Se = 99.02%	Detection: Acc = 96.50% Se = 97.10% Location Acc = 62.94% Se = 63.97%
Multi-scale feature [41]	12 leads PTB	Detection and location	/	Detection: Acc = 95.76% Location: Acc = 61.82%
DenseNet [17]	12 leads PTB	Location	Acc = 99.87% Se = 99.84% Sp = 99.98%	/
Multi-scale ResNet with attention [42]	12 leads PTB	Detection and location	Detection: Acc = 99.98% Se = 99.94% Location: Acc = 99.79% Se = 99.88%	/
3D ECG images [37]	12 leads PTB	Detection	Acc = 100.00% Se = 100.00% Sp = 100.00%	Acc = 95.65% Se = 97.34% Sp = 90.80%
Tucker2 decomposition [43]	12 leads PTB	Location	Acc = 99.67% Se = 99.98% Sp = 99.82%	Acc = 65.11% Se = 98.29% Sp = 71.91%
Multi-lead branch with ResNet with SE and LSTM [44]	12 leads PTB	Detection and location	Detection: Acc = 99.94% Se = 99.99% Location: Acc = 99.69% Se = 99.58%	Detection: Acc = 96.55% Se = 96.17% Location: Acc = 67.89% Se = 63.16%
**MFF–CNN** **(Our)**	**12 leads** **PTB**	**Detection** **and** **location**	**Detection:** **Acc = 99.99%** **Se = 100.00%** **Location:** **Acc = 99.98%** **Se = 99.97%**	**Detection:** **Acc = 99.98%** **Se = 100.00%** **Location:** **Acc = 84.86%** **Se = 62.90%**

## Data Availability

No datasets were generated during the current study.
